# Oncologists' knowledge, attitudes and needs about artificial intelligence in clinical oncology in Luxembourg in 2026: a national cross-sectional survey (AICO study)

**DOI:** 10.3389/fdgth.2026.1860757

**Published:** 2026-08-25

**Authors:** Dominic Kaddu-Mulindwa, Xianqing Mao, Caroline Duhem, Sigrid de Wilde, Stefan Rauh, Gilles Klein, Frank Jacob, Sophie Servais

**Affiliations:** 1 Centre Hospitalier du Nord, Ettelbruck, Luxembourg; 2 Faculty of Science, Technology and Medicine (FSTM), Department of Health, Medicine and Life Sciences, University of Luxembourg, Esch-sur-Alzette, Luxembourg; 3 Department of Hematology-Oncology and Cancer Medicine, Centre Hospitalier de Luxembourg, Luxembourg City, Luxembourg; 4 Centre Hospitalier Emile Mayrisch, Esch-sur-Alzette, Luxembourg

**Keywords:** artificial intelligence, clinical decision support, digital health, ethics, oncology, survey

## Abstract

**Introduction:**

Artificial intelligence (AI) is rapidly entering the field of medical oncology. Clinician literacy, ethical awareness, and governance structures are prerequisites for safe implementation, yet real-world data on adoption, training and ethical perceptions from small European healthcare systems remain scarce.

**Methods:**

We conducted an anonymous national cross-sectional online survey among all practicing physicians (medical oncologists, radiation oncologists, haematologist-oncologists) and oncology residents in Luxembourg (*n* = 42 eligible) between January and February 2026. The instrument covered AI knowledge, use, attitudes, ethical/legal perspectives, barriers and training needs. Proportions are reported with 95% Wilson score confidence intervals; analyses were descriptive.

**Results:**

In total 25 physicians responded (59.5%), 88% (95% CI 70.0–95.8) of whom had no formal AI training. All respondents had used large language models (LLMs), with 52% (33.5–70.0) reporting professional use for non-clinical tasks and 20% (8.9–39.1) for clinical decision support. Most participants (84%; 65.3–93.6) supported the use of AI as a clinical decision-support tool, yet 88% (70.0–95.8) of physicians perceived they would bear primarily legal responsibility for AI-related errors. The main barriers to implementation were lack of validated tools (68%; 48.4–82.8) and regulatory/legal uncertainty (64%; 44.5–79.8). Notably, 76% (56.6–88.5) of respondents reported encountering patients who brought AI-generated medical information to consultations.

**Discussion:**

AI adoption among oncologists in Luxembourg is already widespread, including emerging clinical use, despite limited formal training and unresolved medico-legal frameworks. This “implementation–governance gap” highlights the need for structured education, validated clinical tools, and regulatory clarity to ensure safe and ethically sound integration of AI into oncology practice.

## Introduction

Artificial intelligence (AI) is rapidly entering the field of clinical medicine (1). Medical AI systems fall under the high-risk category of the European Union Artificial Intelligence Act (Regulation (EU) 2024/1689) (9), requiring conformity assessment, transparency, human oversight and post-market monitoring. Over recent years, AI applications, including machine learning algorithms, clinical decision-support systems, and large language models (LLMs), are increasingly embedded in oncology practice (2). International physician surveys report heterogeneous knowledge and adoption patterns but consistently high interest in structured education (3, 4). Studies from several countries demonstrate comparable enthusiasm tempered by concerns regarding validation, bias, and legal responsibility (5–7). To date, however, no data exist for Luxembourg, a small but highly interconnected healthcare system in which a limited number of centres serve the entire national oncology population.

Even though the evidence base underlying clinician attitudes is by now substantial it still remains heterogeneous. A systematic review with cross-sectional survey found broadly favourable acceptance of clinical AI among physicians and medical students, moderated by perceived transparency and trust (4). Among oncologists specifically, Hantel et al. reported strong endorsement of AI-assisted care coupled with pronounced concerns about explainability, informed consent and responsibility for error (3). Surveys in German university hospitals (5) and among Italian radiation oncologists (6) converge on the same asymmetry: high willingness to adopt, low structured preparation. A parallel literature from human-factors research cautions that decision-support systems can induce automation bias, meaning systematic over-reliance on algorithmic output, generating both omission and commission errors, and that the risk is greatest where users lack training in the tool's failure modes (10). Reasoning about liability follows a similar pattern: physicians are widely predicted to remain the most exposed party under existing tort doctrine because standard-of-care reasoning anchors on the clinician rather than the tool (11). Taken together, these strands motivate examining not just whether oncologists use AI, but whether the surrounding educational and governance scaffolding has kept pace.

Luxembourg offers an instructive setting. The Grand Duchy has a statutory health-insurance system with universal coverage, free choice of provider and no gatekeeping, and its care delivery is markedly hospital-centric and highly centralised; even though there is an emergent training program for resident oncologists since a couple of years, most of the physician workforce is heavily trained abroad, which imports heterogeneous professional norms (12). Oncology care is concentrated in four acute-care hospital centres together with the national radiotherapy center, and is coordinated nationally by the *Institut National du Cancer* under successive National Cancer Plans, the second of which places explicit emphasis on digitalisation and data exchange (13). Approximately 3,000 new cancers are diagnosed annually in a resident population of roughly 670,000. Consequently, the entire national oncology physician workforce is small enough to be enumerated rather than sampled—the premise of the present census—and any governance decision propagates quickly across the system. These features make Luxembourg a tractable natural laboratory for questions of AI implementation and oversight in small EU health systems.

This study aimed to assess oncologists' knowledge, use, attitudes, and perceived barriers regarding AI in Luxembourg in 2026, and to characterise the distance between observed adoption and the governance structures intended to regulate it.

## Methods

We conducted a national cross-sectional anonymous online survey between January and February 2026. All practicing physicians (medical oncologists, radiation oncologists, haematologist-oncologists) and residents in medical oncology in Luxembourg, from both hospital and private practice, were invited to participate via national professional mailing lists (whole-population/census approach). Participation was voluntary and anonymous. Two reminders were sent at weeks 2 and 4.

The survey was administered using Microsoft Forms, selected because it is covered by the institutional Microsoft 365 data-processing agreement, hosts data within the European Union, and was already approved for institutional use under the General Data Protection Regulation (GDPR). No directly identifying information was collected, IP-address logging was disabled, and responses were stored on an access-restricted institutional tenant. Because responses were fully anonymous, uniqueness of response could not be technically enforced; the closed distribution list, the invitation to a small, well-defined national population, and the short field period were used to minimise the risk of duplicate submissions.

Items of the survey were adapted from previously published physician AI surveys (5–7); item stems and response options were translated, and the domain structure (knowledge, use, attitudes, ethics/legal, barriers, training) was retained. The instrument was not formally re-validated; face and content validity were assessed through expert review by two senior oncologists and pilot-testing for clarity. The questionnaire comprised five domains: demographics; AI knowledge; AI use; ethical/legal perspectives; and barriers and training needs. Regarding the definition of clinical AI use, no predefined operational criteria were provided to distinguish between “clinical decision support” and “professional use for non-clinical tasks”. Respondents classified their own AI use according to their interpretation of these categories. Self-classification was not independently verified. The full instrument is provided as Supplementary File S1.

Attitudinal items used five-point Likert scales (1 = strongly disagree to 5 = strongly agree). For the dichotomised proportions reported here, responses of 4 (“agree”) or 5 (“strongly agree”) were collapsed into “agreement/support” and responses of 1–3 into “neutral/disagreement”. Barriers were assessed with a single item offering a fixed list of pre-specified options (lack of training, lack of validated tools; data privacy concerns, integration with workflow, cost, lack of trust, legal uncertainty), with multiple selections permitted and an optional free-text “other” field.

The study is reported in accordance with the Checklist for Reporting Results of Internet E-Surveys (CHERRIES) (14). A completed, item-by-item CHERRIES checklist is provided as Supplementary File S2, covering survey design, ethics and consent, development and pre-testing, recruitment and survey administration, and response-rate definitions.

Categorical variables are summarised as counts and percentages, with 95% Wilson score confidence intervals. *Post-hoc* exploratory subgroup summaries were performed for descriptive purposes only. No formal hypothesis testing was planned or performed, and these analyses should be considered hypothesis-generating. Analyses were performed in R version 4.4 (R Foundation for Statistical Computing, Vienna, Austria). Figures were prepared with GraphPad Prism (GraphPad Software, San Diego, CA, USA).

Demographic questions captured age band, gender, primary specialty, practice setting and years of post-qualification clinical experience. Gender was recorded with the response options “male”, “female” and “prefer not to disclose” the instrument therefore did not impose a binary classification, and no respondent selected “other”.

The study was performed in line with the Declaration of Helsinki and was reviewed and approved by the Ethics Review Panel of the University of Luxembourg. All participants provided informed consent.

## Results

Of 42 eligible physicians, 25 responded (59.5%). Respondents' characteristics are shown in Table 1. Respondents were predominantly aged 23–45 years (76%) and male (56%). Most were medical oncologists (60%) working in hospital settings (76%).

**Table 1:** Respondents' characteristics (*n* = 25).

**Characteristic**	***n* (%)**
Age 23–35 years	8 (32%)
Age 36–45 years	11 (44%)
Age ≥46 years^a^	6 (24%)
Male	14 (56%)
Female	10 (40%)
Prefer not to say/not disclosed	1 (4%)
Years since qualification: <5	(8) (32%)
Years since qualification: 5–10	(6) (24%)
Years since qualification: ≥10	(11) (44%)
Medical oncologist	15 (60%)
Haematologist	4 (16%)
Radiation oncologist	2 (8%)
Oncology fellow	2 (8%)
Hospital practice	19 (76%)
Hospital + private	6 (24%)

a Age categories ≥46 years were grouped for analysis owing to small subgroup sizes. Gender was self-reported from the options male/female/prefer not to say. Years since qualification refers to years of post-qualification clinical practice.

The median completion time of the survey was 8 min 56 s. All key proportions with 95% confidence intervals are summarised in Table 2.

**Table 2:** Key survey findings with 95% confidence intervals (*n* = 25).

**Survey item**	**n/N**	**% (95% CI)** ^a^
No formal AI training	22/25	88 (70.0–95.8)
Used LLMs (any purpose)	25/25	100 (86.7–100)
Professional use—non-clinical tasks	13/25	52 (33.5–70.0)
Professional use—clinical decision support	5/25	20 (8.9–39.1)
No professional use (personal only)	7/25	28 (14.3–47.6)
Support AI as clinical decision-support tool	21/25	84 (65.3–93.6)
Perceive primary legal responsibility for AI errors	22/25	88 (70.0–95.8)
Clinicians should participate in AI development	21/25	84 (65.3–93.6)
Interest in structured AI education	19/25	76 (56.6–88.5)
Encountered patients bringing AI-generated information	19/25	76 (56.6–88.5)
Barrier: lack of validated tools	17/25	68 (48.4–82.8)
Barrier: regulatory and legal uncertainty	16/25	64 (44.5–79.8)
Barrier: lack of trust in AI outputs	13/25	52 (33.5–70.0)
Barrier: data privacy and security concerns	12/25	48 (30.0–66.5)

a Percentages with 95% Wilson score confidence intervals. Barrier items allowed multiple selections; perce*n*tages therefore do not sum to 100%. AI, artificial intelligence; CI, confidence interval; LLM, large language model.

**Figure 1:**
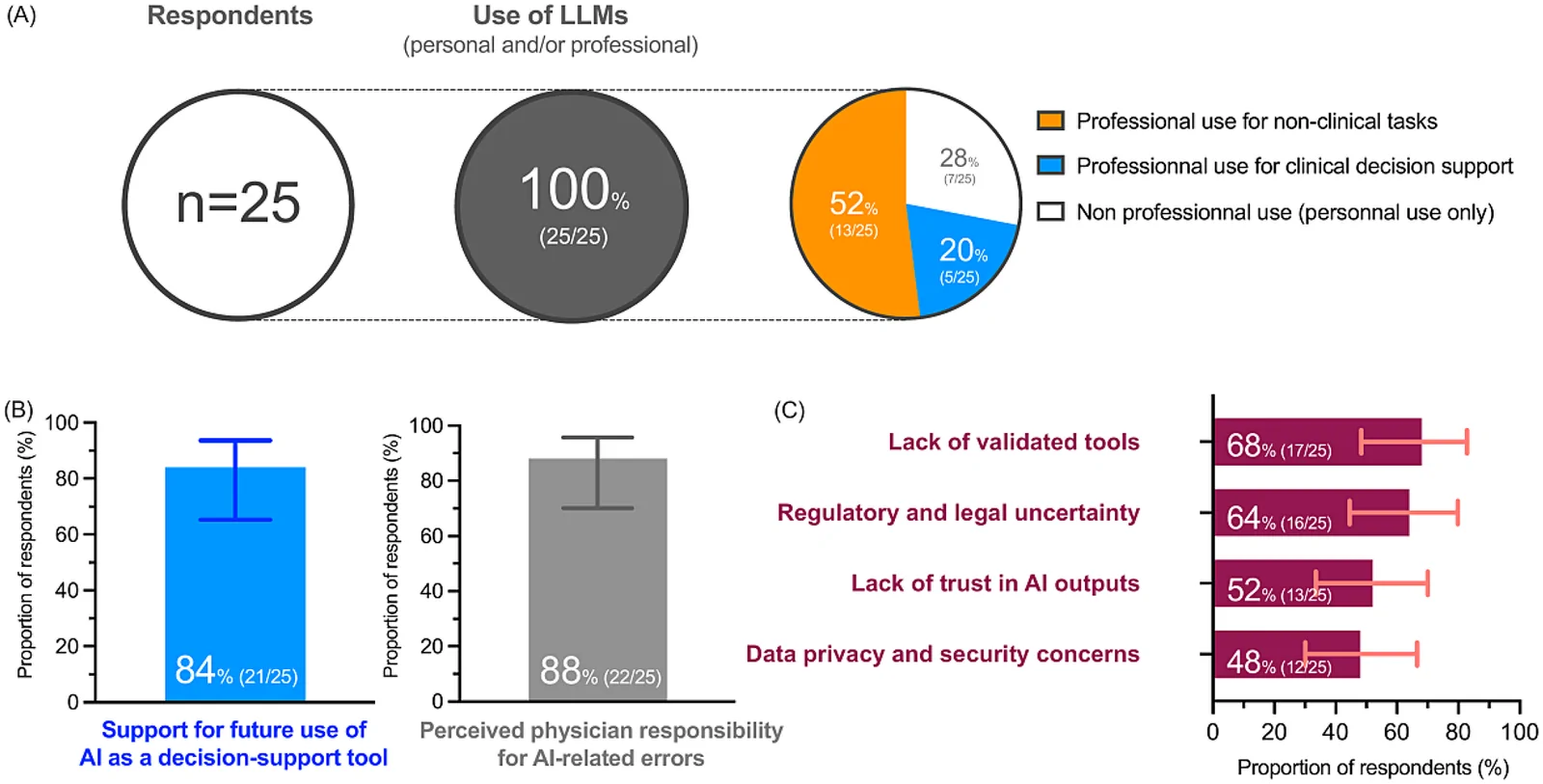
AI use **(A)**, attitudes **(B)** and perceived barriers **(C)** among oncologists in Luxembourg in 2026 (*n* = 25 respondents). **(A)** Universal use of large language models (100%) and distribution of professional AI use across all respondents (denominator *n* = 25): non-clinical tasks 52%, clinical decision support 20%, and personal use only 28%. **(B)** Support for AI as a decision-support tool and perceived physician responsibility for AI-related errors. **(C)** Perceived barriers to implementation; multiple selections were permitted, so percentages do not sum to 100%. Error bars in panels **(B)** and **(C)** denote 95% Wilson score confidence intervals.

In total, 88% of participants (95% CI 70.0–95.8) reported no prior formal AI training; equivalently, only 12% (4.2–30.0) had received any formal training (Figure 1A). All respondents (100%; 86.7–100) reported using AI systems—especially LLMs— for personal and/or professional purposes. Among professional users, use was primarily for non-clinical tasks (52%; 33.5–70.0), whereas 20% (8.9–39.1) reported use for clinical decision support and 28% (14.3–47.6) reported personal use only (Figure 1A).

More than three-quarters of participants (84%; 65.3–93.6) considered that AI should be used as a supportive tool in medical decision-making but, yet most (88%; 70.0–95.8) perceived they would still bear primarily legal responsibility for AI-related errors (Figure 1B). Overall, 84% (65.3–93.6) agreed clinicians should participate actively in AI development, and 76% (56.6–88.5) expressed interest in structured AI education. Notably, 76% (56.6–88.5) reported encountering patients who brought AI-generated medical information to consultations (Table 2). Perceived barriers are summarised in Figure 1C; the two leading barriers were lack of validated tools (68%; 48.4–82.8) and regulatory/legal uncertainty (64%; 44.5–79.8).

### Post-hoc exploratory descriptive subgroup analyses

Among physicians reporting clinical use of AI (*n* = 5), all expressed confidence in AI as a supportive decision-making tool (100%, 95% CI 56.5–100), compared with 80% (58.4–91.9) of those reporting exclusively non-clinical or no professional AI use (*n* = 20). Similarly, professional AI use was reported by 79% (56.7–91.5) of younger physicians (<46 years, *n* = 19) and by 50% (18.8–81.2) of older physicians (≥46 years, *n* = 6). Given the small subgroup sizes, these exploratory analyses were not designed or powered to support between-group comparisons and should be considered hypothesis-generating only. Sample sizes for other exploratory subgroup summaries (e.g., according to specialty or years of experience) were considered too small to allow meaningful interpretation and are therefore not presented.

## Discussion

This national survey from Luxembourg reveals a striking paradox: near-universal exposure to generative AI tools despite minimal structured education. We use the term “implementation–governance gap” to denote the distance between the de-facto uptake of AI in routine clinical work (implementation) and the maturity of the training, validation, liability and oversight structures intended to make that use safe and accountable (governance). This framing parallels the long-standing “know–do gap” in implementation science and the JAMA Summit's call for governance structures to keep pace with clinical deployment of AI (1), and it is directly relevant to small, densely interconnected health systems where diffusion can outpace formal oversight.

Similar patterns of enthusiasm coupled with limited structured training have been reported in other European settings (5, 6). These comparisons should, however, be drawn with attention to differences in sample composition. The Italian study by Piras et al. (6) specifically examined radiation oncologists' use of ChatGPT within a single professional society (yAIRO), a narrower population and a single-tool focus, whereas our census captured medical, radiation and haematological oncology across all national providers and multiple AI applications. The Brazilian study by Giavina-Bianchi et al. (7) surveyed a broader, non-oncology-specific physician population and, like our cohort, reported optimism regarding AI's diagnostic potential alongside pronounced medico-legal uncertainty. Thus, while the direction of attitudes is consistent across settings, our findings extend them to an entire national oncology workforce and to a period (2026) of active EU AI Act implementation. The primary barriers for oncologists in Luxembourg, lack of validated tools and legal uncertainty, highlight the need for structured implementation pathways. Therefore, education initiatives should include AI literacy, risk assessment, bias awareness, and regulatory compliance.

A key observation is the discrepancy between AI adoption and perceived responsibility. Although most participants supported AI as a clinical decision-support tool, nearly all believed that physicians would retain primary legal responsibility for AI-related errors. This perception contrasts with the allocation of obligations under the EU AI Act, which distributes duties across providers and deployers of high-risk systems: providers must satisfy conformity assessment, data governance, logging and post-market monitoring requirements, and must design systems for effective human oversight (Article 14), while a deployer that substantially modifies or rebrands a system may itself assume provider obligations (Article 25) (9). The two dominant barriers we identified map directly onto this framework: the perceived lack of validated tools reflects the conformity-assessment and post-market-monitoring duties that providers must discharge before trustworthy tools reach the clinic, while legal uncertainty reflects the unsettled allocation of civil liability discussed below.

Reflecting further, the respondents' perception may be closer to the legal reality than the “shared responsibility” framing suggests, and this deserves emphasis rather than reassurance. Two distinctions matter. First, the AI Act allocates regulatory obligations under public law; it does not itself create a civil cause of action or reallocate fault between clinician and manufacturer when a patient is injured. Second, the instrument that would have eased claimants' burden of proof against AI providers—the proposed AI Liability Directive—was listed for withdrawal in the Commission's 2025 Work Programme and formally withdrawn in October 2025 (15) leaving fault-based claims arising from AI-assisted care to the national tort law of each Member State. What survives is the revised Product Liability Directive (EU) 2024/2853, which extends strict liability to software, including AI systems, but is principally directed at producers rather than professional users and must be transposed by December 2026 (16). The net effect for a clinician in Luxembourg in 2026 is that liability remains predominantly fault-based and clinician-anchored, because the standard of care continues to be defined by professional practice rather than by the tool (11). Our respondents' near-unanimous expectation of primary responsibility is therefore not a misconception to be corrected by education alone; it is a rational reading of an incomplete governance architecture.

This reframing has practical consequences. If clinicians correctly anticipate residual personal exposure, then trust in AI cannot be manufactured by asserting shared responsibility; it must be earned through validated tools, auditable deployment and institutional risk-sharing. It also sharpens the risk of automation bias: a clinician who both bears the liability and lacks training in a system's failure modes is precisely the user in whom over-reliance is most consequential (10). Documentation-oriented LLM use, reported by one in five of our respondents, is often perceived as low-risk, yet errors propagate silently into the record and downstream decisions. Concrete mitigations—mandatory human verification of AI-generated documentation, local logging of tool use, institutional indemnity arrangements, and inclusion of AI outputs in existing morbidity-and-mortality review—would translate the abstract governance debate into mechanisms a small national system such as Luxembourg could realistically implement, and could plausibly be piloted through existing national coordination structures (13).

The persistence of physician-centered liability perceptions may foster hesitancy, defensive practice, or inconsistent adoption, and align with the ethical concerns oncologists have voiced elsewhere regarding accountability and transparency (3) Luxembourg's small healthcare ecosystem may facilitate rapid, coordinated implementation but simultaneously magnifies liability and trust concerns, as a small number of decisions and centers shape national practice.

The finding that 84% of respondents felt clinicians should participate actively in AI development is an important and constructive counterpoint to these concerns: oncologists are not merely passive recipients of AI but seek a role in shaping tools, training data and governance. This appetite for co-design is a practical lever for closing the implementation–governance gap and is consistent with clinician-led guidance such as the ESMO recommendations on the use of LLMs in clinical practice (ELCAP) (2) which provide a concrete, oncology-specific framework covering appropriate use cases, verification of outputs, data protection and documentation. Embedding ELCAP-style guidance into local governance and continuing medical education would give the 76% of respondents requesting structured training a validated starting point, and structured educational supervision models such as that proposed by Abdulnour et al. (8) offer a template for integrating AI use into clinical training and oversight.

That 76% of oncologists reported patients arriving with AI-generated medical information underscores that AI is already embedded in the daily clinical encounter, independent of physician adoption. This shifts part of the oncologist's role toward interpreting, contextualising and, where necessary, correcting AI-generated content—an emerging “sense-making” task with implications for consultation length and dynamics, shared decision-making, and health literacy. It also creates a concrete, patient-facing rationale for AI literacy training that extends beyond the physician's own tool use. The parallel finding that one in five respondents already use LLMs for clinical decision support—most often for documentation—further highlights unresolved questions of data privacy and medico-legal accountability that current governance has yet to answer.

### Strengths and limitations

Strengths include the whole-population (census) design, which removes sampling-based selection bias, and a response rate (59.5%) that is satisfactory for physician surveys and represents a substantial fraction of the national oncology workforce. Limitations include the small absolute sample size (*n* = 25), which yields wide confidence intervals, and the descriptive study design. Accordingly, no formal hypothesis testing was planned or performed, and the exploratory subgroup analyses were not powered and are hypothesis-generating only. As with all survey research, results are subject to self-report and potential response bias, possibly favoring clinicians with greater interest in digital technologies. Finally, the classification of “clinical” vs. “non-clinical” AI use relied on respondent self-categorisation and was not independently verified; misclassification could shift the 20% clinical-use estimate in either direction, although the broader conclusion of early, real-world clinical adoption is robust to modest misclassification. Because the survey was anonymous, non-respondents could not be characterised and no formal non-response analysis was possible; the 40.5% who did not reply may differ systematically in AI engagement, and the estimates reported here should be read as describing the responding subgroup rather than the workforce with certainty. The study is also cross-sectional, capturing attitudes during a period of active EU AI Act implementation, so perceptions of liability in particular may shift as national case law develops. Findings from a single small health system may not generalise to larger or more decentralised systems, although the mechanisms we describe—adoption preceding governance, and clinician-anchored liability—are not specific to Luxembourg.

In conclusion, oncologists in Luxembourg demonstrate high engagement with AI tools, including early clinical use, despite limited formal training and unresolved legal frameworks. Bridging this implementation–governance gap will require coordinated efforts in education, validation, and regulation to ensure that AI integration into oncology practice is safe, effective, and ethically grounded.

## Data Availability

The raw data supporting the conclusions of this article will be made available by the authors, without undue reservation.
