# Supplementary material for: Oncologists' knowledge, attitudes and needs about artificial intelligence in clinical oncology in Luxembourg in 2026: a national cross-sectional survey (AICO study)
Source: Front Digit Health. 2026 Aug 25;8:1860757. doi: 10.3389/fdgth.2026.1860757 (PMC13550199; doi:10.3389/fdgth.2026.1860757)
Supplement: Supplementary file 1 [file Supplementaryfile1.docx]

**Supplementary File S1. Survey instrument (AICO study).**

**Online survey / questionnaire**

**Page 1: Consent & intro**

Title: AI in Oncology — survey for Luxembourg oncologists

“You are invited to participate in an anonymous survey of oncologists in Luxembourg about knowledge, use and attitudes towards artificial intelligence (AI) in oncology. Estimated time: 6–8 minutes. Participation is voluntary and anonymous. Results will be used for research and local training planning. By ticking ‘I agree to participate’ you acknowledge that your anonymized responses collected when submitting the survey will be used for research. To mitigate the theoretical reputational risk from AI misuse, no raw survey data will be processed by external AI tools and we will conduct all analysis on secure institutional servers. Free-text responses will be manually screened to remove any potentially identifying information before analysis. Results will only be reported in aggregated form. Data access is strictly limited to the research team and no raw qualitative data will be published. These measures eliminate the risk that AI tools could re-identify or harm participants.”

Q1 (required, single choice) I agree to participate in the survey*.

• Yes

**Page 2: Demographics & practice**

**In the open text session, please do not provide any personal data in this field.**

Q2 (single choice) — Your age bracket (years)*.

- 23–35 years
- 36–45 years
- 46–55 years
- 56–65 years
- More than 65 years

Q3 (single choice) — Gender: Female / Male / Prefer not to disclose / Other*.

Q4 (short choice) — Years since qualification (practising medicine)*.

- Less than 5 years
- 5 - 10 years
- 10 - 20 years
- 20 – 30 years
- More than 30 years

Q5 (short choice) — Years working in oncology*.

- Less than 5 years
- 5 - 10 years
- 10 - 20 years
- 20 – 30 years
- More than 30 years

Q6 (single choice) — Primary clinical role: Medical oncologist / Radiation oncologist / Surgical oncologist / Haematologist / Oncology fellow / Other (text)*.

Q7 (single choice) — Workplace type: Hospital / Private practice / Both*.

**Page 3: Training & exposure to AI**

**In the open text session, please do not provide any personal data in this field.**

Q8 (single choice) — Have you received any formal education/training in AI*?

• No training / Basic / Intermediate / Advanced.

Q9 (multiple choice) — Which AI systems or tools have you used*? (choose all that apply)

• I have not used any

• ChatGPT or other Large language models (e.g., GPT models)

• Clinical decision support (CDS) / knowledge-based CDS tools

• Imaging AI applications (CT/MRI/pathology)

• Electronic health record embedded AI features (e.g., note autocompletion)

• Other (text).

Q10 (single choice) — Have you used ChatGPT (or similar large language model) for professional tasks*?

• Never / Only personally / For non-clinical tasks (writing, admin) / For clinical tasks (note: specify in free text).

Q11 (single choice) — Would you like more education/training on AI*? Yes / No / Unsure.

**Page 4: Self-rated knowledge (Likert 1–5)**

Instruction: Rate your level of knowledge for each (1 = No knowledge / 5 = Very well-informed).

Q12*

Large Language Models (LLMs) — 1–5

Machine Learning — 1–5

Deep Learning — 1–5

Neural networks — 1–5

Natural Language Processing — 1–5

Generative AI — 1–5

Basic understanding of algorithmic bias / limitations — 1–5

**Page 5: Attitudes & perceived usefulness (Likert)**

Instruction: For each domain, choose: Very negative / Negative / Undecided / Positive / Very positive. (adapted).

Q13 Use of AI for diagnosis (imaging/path report triage)* — 5-point scale

Q14 Use of AI for treatment planning (radiation planning / therapy selection support)* — 5-point scale

Q15 Prognosis estimation / risk prediction* — 5-point scale

Q16 Clinical decision support at point of care* — 5-point scale

Q17 Patient follow-up / remote monitoring* — 5-point scale

Q18 Research & data analytics* — 5-point scale

Q19 (single choice) — Do you think AI should be used as a supportive tool in medical decision-making*? Yes / No / Undecided.

Q20 (single choice) — Do you think clinicians should participate actively in AI development*? Yes / No / Undecided.

**Page 6: Ethical / legal concerns**

Instruction: Rate level of concern: No concern / Slight concern / Concern / High level of concern / Very high concern.

Q21 Patient privacy / data protection* — 5-point

Q22 Reliability of AI outputs / clinical safety* — 5-point

Q23 Transparency / black-box issues* — 5-point

Q24 Algorithmic bias / fairness* — 5-point

Q25 Authorship & use of AI in publications* — 5-point

Q26 Impact on physician-patient relationship / empathy* — 5-point

Q27 (multiple choice) — In which activities do you consider AI ethically questionable*? (tick all that apply)

• Study design / protocol writing

• Article writing / editing (authorship concerns)

• Ethics committee applications

• Direct patient management (clinical decisions)

• Communication materials for patients

• None of these / Other (text).

Q28 (multiple choice) — In the event of an AI-related medical error, who should bear legal responsibility*? (select all that apply)

• Physician / Software developer / Healthcare institution / AI trainer / Patient (if consented) / Other.

Q29 If you choose other as option in the previous question, please share here.*

(open text)

**Page 7: Barriers, enablers & training preferences**

Q30 (multiple choice) — What are the main barriers to adopting AI in oncology in your setting*? (choose up to 3)

• Lack of training / Lack of validated tools / Data privacy concerns / Integration with workflow / Cost / Lack of trust / Legal uncertainty / Other (text).

Q31 If you choose other as option in the previous question, please share here.*

(open text)

Q32 (multiple choice) — Which measures should be taken to ensure ethical AI*? (choose all that apply)

• Legal regulation / Transparent algorithms / Obtaining patient consent / Widespread clinician training / Ethical audits / National working groups / Other (text).

Q33 (single choice + follow-up) — Which training format would you prefer*?

• Short practical workshop (2–4 h) / Online self-paced course / Half-day seminar / Continuous medical education series / One-to-one consultancy / Other (text).

**Page 8: Future outlook & free text**

Q34 (5-point scale) — How do you think AI will affect* (Very negative → Very positive): medical practice / oncologists’ role / access to care / workload / job satisfaction. (adapted).

Q35 (open text) — What is your greatest concern or priority regarding AI ethics? (optional).

Q36 (open text) — What do you think are the biggest challenges for development/implementation of AI in oncology in Luxembourg? (optional).

Q37 (open text) — Suggestions for integrating AI ethically and effectively (optional).

Q38 (single choice) — Have you ever had a patient who brought AI-generated information about their disease to a consultation with you*? yes / no

Q39 (final optional) — Any other opinions or experiences regarding AI & oncology? (optional).

**End page**

“Thank you.
