# Supplementary material for: Oncologists' knowledge, attitudes and needs about artificial intelligence in clinical oncology in Luxembourg in 2026: a national cross-sectional survey (AICO study)
Source: Front Digit Health. 2026 Aug 25;8:1860757. doi: 10.3389/fdgth.2026.1860757 (PMC13550199; doi:10.3389/fdgth.2026.1860757)
Supplement: Supplementary file 2 [file Supplementaryfile2.docx]

**Supplementary File S2. CHERRIES checklist (AICO study).**

*Checklist for Reporting Results of Internet E-Surveys (CHERRIES; Eysenbach, J Med Internet Res 2004). Item-by-item description of how the AICO survey was designed, administered and reported. Section references are to the main manuscript.*

| **Category / Item** | **Description for this study** |
| --- | --- |
| **Design** | National cross-sectional, anonymous online survey conducted as a whole-population census of all 42 physicians eligible to practise clinical oncology in Luxembourg (medical, radiation and haematologist-oncologists and oncology residents). Fielded January–February 2026 (Methods). |
| **IRB approval** | Reviewed and approved by the Ethics Review Panel of the University of Luxembourg (Ethics section). |
| **Informed consent** | A consent statement preceded the questionnaire; participation was voluntary and constituted consent. No directly identifying information was collected. |
| **Data protection** | Administered via Microsoft Forms under the institutional Microsoft 365 data-processing agreement, with data hosted in the EU and prior GDPR approval for institutional use. IP-address logging was disabled and responses were stored on an access-restricted institutional tenant (Methods). |
| **Development and testing** | Items were adapted from previously published physician AI surveys [5,6,7], retaining their domain structure across five domains (demographics; AI knowledge; AI use; ethical/legal perspectives; barriers and training needs). The instrument was not formally re-validated; face and content validity were assessed by expert review by two senior oncologists and pilot-testing for clarity (Methods; full instrument in Supplementary File S1). |
| **Recruitment process** | Closed survey. Invitations were distributed through national professional mailing lists to the entire eligible population; the questionnaire was not openly advertised or publicly accessible. Two reminders were sent (weeks 2 and 4). |
| **Survey administration — context** | Professional survey of practising oncologists; not linked to any patient care or institutional obligation. Participation was voluntary. |
| **Survey administration — mandatory/optional items** | Response options included “prefer not to disclose” / free-text “other” where appropriate; items were not forced. |
| **Survey administration — adaptive questioning** | Yes. Conditional (adaptive) questioning was used, so the number of items shown depended on earlier responses. |
| **Survey administration — number of items and pages** | 39 questions presented across 8 screens/pages, preceded by the consent page (Supplementary File S1). |
| **Survey administration — back button** | Enabled; respondents could review and change previous answers before submission. |
| **Response rates — participation/completion rate** | 25 of 42 eligible physicians responded (59.5%). All 25 returned questionnaires were usable and included in the analysis. |
| **Response rates — view/visit rate** | Not applicable/available: as a closed survey distributed by direct invitation, unique visitors to a landing page were not tracked, so a view rate could not be computed. |
| **Response rates — completion time** | Median time to complete the questionnaire was 8 min 56 s. |
| **Preventing multiple entries — cookies / IP / log-file** | No cookies were set, IP addresses were not logged, and no log-file or registration-based de-duplication was performed (consistent with full anonymity). Because responses were anonymous, uniqueness of response could not be technically enforced. |
| **Preventing multiple entries — mitigation** | Risk of duplicate submissions was minimised by the closed single-use distribution list, the small well-defined national population, and the short field period (Methods). |
| **Analysis — handling of incomplete questionnaires** | All submitted questionnaires were complete and analysed; no imputation was required. |
| **Analysis — statistical methods** | Descriptive analysis only. Categorical variables are summarised as counts and percentages with 95% Wilson score confidence intervals. No formal hypothesis testing was performed; exploratory subgroup summaries are descriptive and hypothesis-generating. Analyses used R version 4.4; figures were prepared with GraphPad Prism (Methods). |
